# The Scleroderma Patient-centered Intervention Network for conducting large-scale international trials of nonpharmacological interventions in scleroderma: a way ahead for lupus?

**DOI:** 10.1186/ar4630

**Published:** 2014-09-18

**Authors:** Brett D Thombs

**Affiliations:** 1 McGill University and the Lady Davis Institute for Medical Research of the Jewish General Hospital, Montréal, QC, Canada

## Background

Psychosocial and rehabilitation interventions are increasingly used to attenuate disability and improve health-related quality of life (HRQL) in chronic diseases, but are typically not available for patients with less common diseases such as scleroderma or lupus. Conducting rigorous, adequately-powered trials of nonpharmacological interventions for patients with rare diseases is difficult. The Scleroderma Patient-centered Intervention Network (SPIN) is an international collaboration of patient organizations, clinicians, and researchers. The aim of SPIN is to develop a research infrastructure to test accessible, low-cost self-guided online interventions to reduce disability and improve HRQL for people living with scleroderma. Once tested, effective interventions will be made accessible through patient organizations partnering with SPIN.

## Methods

SPIN will utilize a novel research design, the cohort multiple randomized controlled trial (cmRCT) design, to collect longitudinal data related to problems experienced by people living with scleroderma and as a framework for developing, evaluating, and delivering psychosocial and rehabilitation interventions. In the cmRCT design, patients consent to participate in a cohort for ongoing data collection. The aim is to recruit 1,500 to 2,000 patients from centers across the world within a period of 5 years (2014 to 2018). Eligible participants are persons ≥18 years of age with a verified diagnosis of scleroderma. In addition to baseline medical data, participants will complete patient-reported outcome measures every 3 months. Upon enrolment in the cohort, patients will consent to be contacted in the future to participate in intervention research and to allow their data to be used for comparison purposes for interventions tested with other cohort participants. Once interventions are developed, patients from the cohort will be randomly selected and offered interventions as part of pragmatic RCTs. Outcomes from patients offered interventions will be compared with outcomes from trial-eligible patients who are not offered the interventions.

## Discussion

The use of the cmRCT design, the development of self-guided online interventions, and partnerships with patient organizations will allow SPIN to develop, rigorously test, and effectively disseminate psychosocial and rehabilitation interventions for people with scleroderma. A similar approach could be used to develop and test psychosocial and rehabilitation interventions for people with lupus.

